# New Strategy for Microbial Corrosion Protection: Photocatalytic Antimicrobial Quantum Dots

**DOI:** 10.3390/nano15010002

**Published:** 2024-12-24

**Authors:** Shijia Liu, Dapeng Wu, Jie Zheng, Baochen Han, Jian Qi, Fanchun Meng, Jianhui Li, Dan Liu

**Affiliations:** 1 Hebei Short Process Steelmaking Technology Innovation Center, School of Materials Science and Engineering, Hebei University of Science and Technology, Shijiazhuang 050018, China; m13224267808@163.com (S.L.); 13485451766@163.com (D.W.); zhengjie200108@163.com (J.Z.); hbchebust@163.com (B.H.); lijianhui_97@163.com (J.L.); 2 State Key Laboratory of Biochemical Engineering, Institute of Process Engineering, Chinese Academy of Sciences, Beijing 100049, China; 3 State Key Laboratory of Coal Conversion, Institute of Coal Chemistry, Chinese Academy of Sciences, Taiyuan 030001, China

**Keywords:** photocatalysis, quantum dots, antimicrobial, microbial corrosion protection

## Abstract

Microbial corrosion has significant implications for the economy, environment, and human safety worldwide. Photocatalytic antibacterial technology, owing to its advantages in environmental protection, broad-spectrum, and efficient sterilization, presents a compelling alternative to traditional antibacterial strategies for microbial corrosion protection. In recent years, photocatalytic quantum dot materials have garnered considerable attention in this field due to their unique quantum effects. This article provides a brief overview of the quantum effects associated with quantum dot materials, reviews the classification and preparation methods of these photocatalytic quantum dots, and elucidates their inhibitory effects and mechanisms against microbial corrosion. Finally, this article summarizes unresolved issues and prospects for the future development of quantum dots in the realm of microbial corrosion protection.

## 1. Introduction

**Figure 1:**
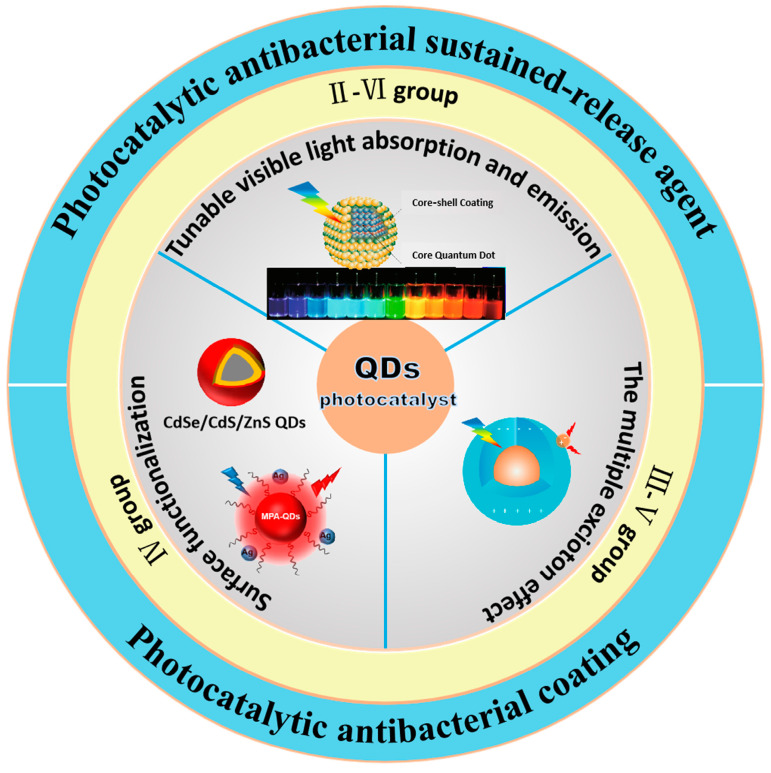
Overview of quantum dot photocatalysts.

Microbial corrosion (MIC), which arises from the biological activities of microorganisms in biofilms that attach to material surfaces, constitutes a major type of corrosion damage to metal substrates. Data show that in 2014, the economic impact of corrosion in China surpassed 2 trillion yuan, representing approximately 3.34% of the GDP [1,2]. Importantly, corrosion caused by microbes accounts for approximately 20% of the overall corrosion. Globally, the financial losses linked to microbial corrosion are estimated to range from 30 to 50 billion US dollars each year [3,4]. This type of corrosion significantly affects industrial productivity as well as the national economy. Typical approaches to mitigate microbial corrosion—including the use of heavy metal coatings [5] and the addition of corrosion inhibitors [6] while somewhat effective, often carry environmental and health hazards [7]. Recently, innovative and sustainable photocatalytic quantum dot materials have surfaced as promising substitutes for traditional anticorrosion methods, thanks to their distinct photocatalytic activity and photoelectric characteristics. Quantum dot materials can improve the photocatalytic efficiency of common photocatalysts (e.g., C_3_N_4_), the main factor often attributed to reduced charge carrier recombination [8]. In addition, quantum dots can act as a response system in self-healing anticorrosion materials, quickly sealing or releasing corrosion inhibitors, while also acting as a precursor to form a protective film on the steel surface [9]. These nanoscale semiconductor materials demonstrate remarkable absorption of visible light, along with multi-exciton effects, surface effects, and size effects, thus offering considerable potential for applications in photocatalysis, especially in antibacterial and anticorrosion domains (Figure 1).

Quantum dot materials represent a category of semiconductor nanomaterials, generally defined by dimensions smaller than 10 nm and notable for their distinct optoelectronic characteristics. Typically, these materials consist of elements from Group II–VI, Group III–V, and Group IV, such as CdS, CdSe, and PbS [10,11,12]. Owing to their minute size and round morphology, quantum dots feature a considerable specific surface area and exhibit colloidal stability in water-based solutions, which greatly affects their interactions across different geometric forms. When the dimensions of a semiconductor material shrink to the molecular level, its physical and chemical traits experience significant transformations as a result of the quantum confinement effect [13]. In the realm of photocatalytic materials, the benefits of quantum dots can mainly be observed across four principal domains.

Quantum Confinement Effect: As the dimensions of semiconductor quantum dots diminish, the conduction band (CB) and valence band (VB) of the semiconductor experience shifts towards more negative and positive energy levels, respectively. This alteration leads to an increase in the optical band gap, which in turn causes luminescence to be characterized by a blue shift in its color [14,15]. For example, decreasing the size of PbSe quantum dots can encompass the full range of luminescence from red to blue [12]. Additionally, when the size of the microstructure approaches either the wavelength of electrons or the Bohr radius of excitons, the behavior of electrons or holes in the system can be perceived as confined within a quantum mechanical potential well. As a result, the initially continuous distribution of energy levels transforms into a discrete set [16].

Quantum Size Effect: As the diameter of a quantum dot semiconductor nears the exciton Bohr diameter, notable alterations occur in its electronic characteristics, demonstrating a quantum size effect. Generally, a majority of the atoms within quantum dots are found concentrated in limited areas on and adjacent to the surface, thereby creating a pronounced interface between the nanocrystal and its surrounding medium. When exposed to light, these nanocrystals reveal incomplete interfaces, which facilitate the generation of electron–hole pairs, consequently impacting their optical properties. The broader implication is that the dimensions of quantum dot particles can be fine-tuned to match the intended energy band structure and spectral absorption range of the semiconductor, thus optimizing the pairing between the conduction band and valence band as well as the electron and hole, ultimately enhancing solar energy utilization [17,18]. Moreover, the minimal distance of photoelectrons from the surface of the material promotes effective charge separation and transfer within quantum dots due to their small size [19,20]. Furthermore, quantum dots demonstrate a multi-exciton effect, where several electron–hole pairs are produced from a single photon excitation, leading to intensified light output [21]. This effect is especially critical for improving the efficiency of solar cells.

Quantum surface effect: It has been observed that when the size of particles reduces, a larger fraction of atoms resides on the surfaces of the nanoparticles, which in turn causes a notable rise in the specific surface area of quantum dots. This occurrence results in poor coordination at the surfaces of the quantum dots, leading to unsaturation. Moreover, the incorporation of keys and hover keys adds to this phenomenon. These exceptionally reactive surface atoms boost the surface energy and activity of the nanocrystals, significantly affecting their optical properties [22,23].

Macroscopic quantum tunneling effect: As the size of nanoparticles decreases and the specific surface area increases, the properties of quantum materials are governed by the principles of quantum mechanics, leading to the manifestation of macroscopic quantum behavior [24,25]. Microscopic particles possess the capability to penetrate barriers. In photocatalytic systems, this specific performance enhances the adsorption of reactants and stabilizes reaction intermediates, thereby lowering the reaction barrier and improving photocatalytic activity [26,27].

**Figure 2:**
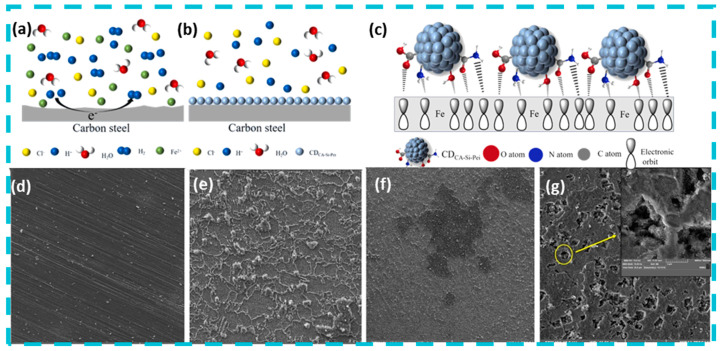
Quantum dots inhibit microbial corrosion. Reprinted with permission from Ref. [30]. Copyright 2023, Elsevier. (**a**) Corrosion mechanism of Q235 carbon steel. (**b**) Corrosion mechanism of CDCA-Si-Pei. (**c**) The adsorption mechanism of carbon steel and CDCA-Si-Pei. The adsorption mechanism of carbon steel and CDCA-Si-Pei. FESEM analysis after removing corrosion products. Reprinted with permission from Ref. [33]. Copyright 2020, Springer Nature. (**d**) Blank coupon. (**e**) SSW. (**f**) Cu/CQDs-SRB. (**g**) SRB. The pits were formed by SRB activity on the surface of X60 steel.

In the field of resistance to microbial corrosion, the application of quantum dot materials has emerged as a practical approach. Several studies indicate that when quantum dot materials are irradiated with visible light, they can rapidly generate reactive oxygen species (ROS). These reactive species can interact with cells, effectively killing a substantial number of bacteria in a short timeframe [28,29]. Notably, some quantum dot materials also serve as innovative, environmentally friendly corrosion inhibitors. For instance, silane functionalized carbon quantum dots, prepared via a hydrothermal method, can bond with iron atoms in carbon steel through oxygen heteroatoms and unsaturated double bonds, thereby forming a protective film on the surface of carbon steel that mitigates damage in acidic environments (Figure 2a–c) [30]. Zhang et al. prepared Sb quantum dots containing Sb, Sb_2_O_3_ and Sb_2_O_4_ by electrochemical stripping method to inhibit the corrosion of Q235 steel. The slow-release efficiency is closely related to the concentration of Sb quantum dots, and reaches the maximum when the concentration of SQDs increases to 200 mg/L [31]. Dong et al. prepared Eco-friendly self-doped carbon quantum dots (ZCQDs) by pyrolysis of Zanthoxylum bungeanum leaves. The prepared ZCQDs contains multiple O and N functional groups, which can form a protective barrier through physical adsorption and chemical adsorption. With the increase in concentration, the protective film formed by ZCQDs will first increase the coverage area on the metal surface, and then adsorb more ZCQDs on the protective film to form a thicker and more dense protective film [32]. Furthermore, the inhibitory effect of quantum dot materials on microbial corrosion can be enhanced by doping with antibacterial metal particles. Sara Taghavi Kalajahi et al. [33] doped carbon quantum dots with copper nanoparticles to create Cu/CQDs nanohybrid, which acts as a sustained-release agent to inhibit microbial corrosion. Experimental results demonstrate that a carbon dot adsorption film forms on the surface of X60, and that Cu/CQDs significantly inhibit sulfate-reducing bacteria (SRB), thereby reducing surface damage to X60 (Figure 2d–g).

**Figure 3:**
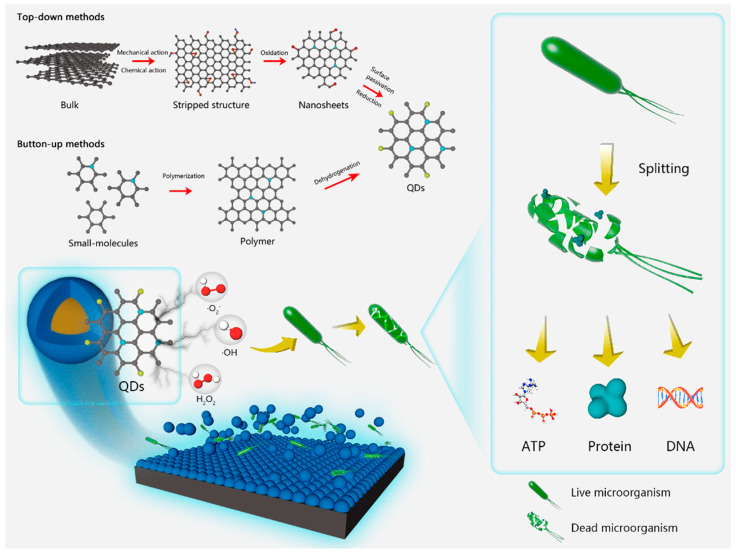
The structure, preparation method, and role in photocatalytic resistance to microbial corrosion of quantum dots.

Given their high activity, straightforward preparation, and surface modification capabilities, quantum dot materials are anticipated to find broader applications in microbial corrosion protection. This article reviews the classification and preparation methods of photocatalytic quantum dot materials, along with their applications in antimicrobial corrosion protection projects. Additionally, it elucidates the inhibitory effects and mechanisms of these materials on microorganisms (Figure 3). Finally, this article summarizes the unresolved issues concerning the application of quantum dots in microbial corrosion protection and offers future prospects.

## 2. Classification and Characteristics of Quantum Dots

In this section, quantum dot materials are broadly classified into three categories according to their elemental composition, group II–VI quantum dots, group III–V quantum dots, and group IV quantum dots.

### 2.1. Group II–VI Quantum Dot Photocatalysts

Group II–VI quantum dot materials represent the earliest and most developed category in quantum dot research. Common examples include cadmium sulfide (CdS), cadmium selenide (CdSe), cadmium telluride (CdTe), and zinc sulfide (ZnS). Since the inception of quantum dots, research on II–VI quantum dots has remained continuous. In 1981, Russian scientist Alexey Ekimov first discovered quantum dots within a glass matrix, employing the potential box model to elucidate the quantum size effect, which describes the relationship between the optical band gap and the size of the nanocrystal [34]. In 1983, Louis Brus confirmed the size effect of CdS nanocrystals by varying their size and named them colloidal quantum dots, thereby officially initiating quantum dot research [35]. In II–VI semiconductors, the valence band and conduction band primarily consist of P orbitals from Group VI elements and S orbitals from metals [36]. Group II–VI semiconductor quantum dots are characterized by their narrow band gap and direct transition band structure, which has garnered significant attention. However, CdS quantum dots exhibit rapid recombination of photogenerated charges and a tendency to agglomerate. Similarly, other metal sulfide quantum dots may experience accumulation of electrons and holes—those that do not participate in the photocatalytic reaction—on the material’s surface under prolonged illumination, leading to participation in redox reactions and consequently resulting in photocorrosion [37]. These phenomena can inhibit the photocatalytic activity of the photocatalyst surface.

**Figure 4:**
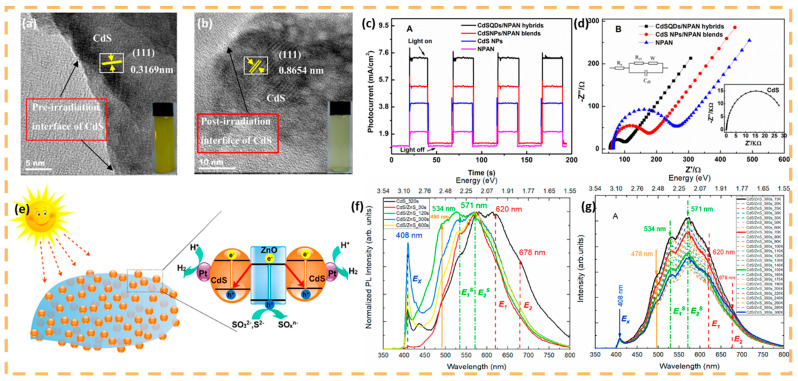
Preparation and modification of quantum dots (**a**,**b**) HRTEM images of pure CdSNPs. Reprinted with permission from Ref. [38]. Copyright 2019, Elsevier. (**c**) Photocurrent response of different electrodes modified by photocatalysts during the light on and light off process. Reprinted with permission from Ref. [38]. Copyright 2019, Elsevier. (**d**) The electrochemical impedance spectra of different-modified electrodes. Reprinted with permission from Ref. [38]. Copyright 2019, Elsevier. (**e**) Proposed mechanism for the photocatalytic hydrogen evolution over the CdS/ZnO heterostructures. Reprinted with permission from Ref. [40]. Copyright 2017, American Chemical Society. (**f**) PL spectra of heterostructures CdS/ZnS and the QD of CdS-320s. (**g**) PL spectra, varying temperature, of heterostructured samples with 300 s of reaction. Reprinted with permission from Ref. [41]. Copyright 2023, Elsevier.2.2. III–V Quantum Dot Photocatalysts.

In recent years, researchers have proposed various solutions to address the photocorrosion issue associated with CdS quantum dots. The primary strategy focuses on mitigating corrosion by enhancing the separation of photogenerated carriers. Functional modifications, such as the introduction of hydroxyl or carboxyl groups, or the doping of quantum dot materials, can partially inhibit the photocorrosion of CdS quantum dots. For instance, Zhang utilized N-substituted carboxyl polyaniline (NPAN) as a precursor to prepare carboxyl-modified polyaniline-modified CdS quantum dots (Figure 4a,b) [38]. The hybridization reaction between CdS quantum dots and carboxyl groups leads to increased photocurrent excitation and enhanced photocatalytic activity (Figure 4c,d). Constructing heterojunctions represents a crucial approach to improving the photocatalytic performance of materials. The energy band bending at the interface of two semiconductors with compatible energy band structures facilitates the separation and transfer of photogenerated carriers, effectively reducing the recombination of these carriers [39]. A heterojunction structure is formed from zero-dimensional CdS quantum dots and two-dimensional ZnO nanosheets (Figure 4e). This Z-shaped heterojunction structure significantly enhances the separation and migration of photogenerated carriers, while the small size effect diminishes the recombination probability of electrons and holes [40]. Building upon this, a core–shell structure can be developed to protect the core from photocorrosion. This coating reduces surface defects and suppresses non-radiative recombination. Everton employed a hot injection method to grow a ZnS shell on CdS quantum dots (QDs), thereby constructing a CdS/ZnS heterostructure. The chemical route utilized effectively controls the growth and enhances the physical and chemical stability of the QDs [41]. Experimental results indicate that, in comparison to quantum dots (QDs), the constructed core–shell heterostructure exhibits reduced surface vacancy defect peaks and enhanced intensity of exciton excitation peaks in the photoluminescence spectrum, with the photoluminescence quantum efficiency rising to 64% (Figure 4f,g). However, it is important to note that various interactions—including crystal structure, electronic state, and energy band structure—occur at the interface of the heterostructure, which compromises its stability. Furthermore, as the temperature increases, the photoluminescence intensity of the core–shell heterostructure tends to decrease.

III–V quantum dots mainly include indium phosphide (InP), indium arsenide (InAs), and gallium phosphide (GaP). Most of the III–V materials are direct band gap semiconductors and do not contain toxic heavy metal elements. III–V quantum dots are ideal for replacing the classical Cd/Pb-based quantum dots, and have a wide range of applications in the field of photocatalytic microbial resistance [42]. However, III–V quantum dots are difficult to prepare due to their stronger covalency [43]. InP quantum dots are a typical type of III–V quantum dots with low toxicity, high absorption coefficient, and high luminescence efficiency, making them a promising photocatalytic material. However, due to its defect-rich structure, for example, the defective states caused by surface dangling bonds will trap electrons and deteriorate the optical properties of quantum dots; a large number of point defects or impurity defects will introduce new defect energy levels within the bandgap; and dislocation defects formed during the oxidation process will form a stress at the oxide-quantum dot interface, which will promote the diffusion of In from the nucleus to the surface of quantum dots, leading to the fluorescence burst phenomenon [44]. These defects hinder the further application of InP quantum dots in photocatalysis.

**Figure 5:**
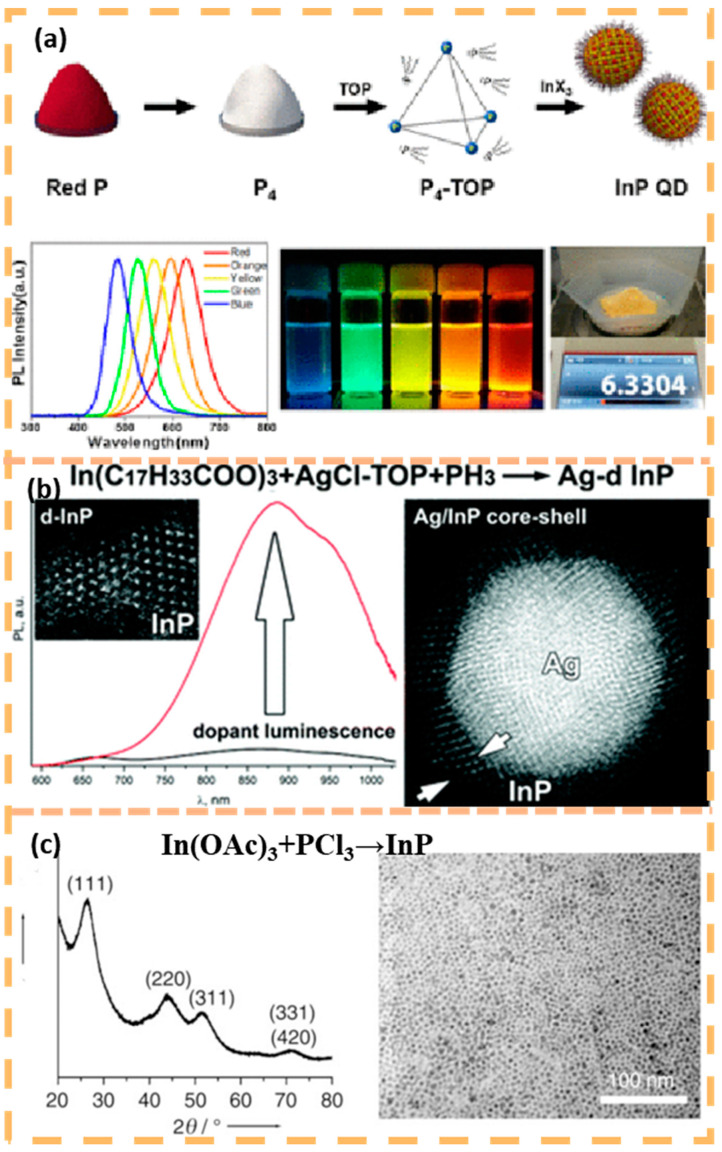
InP quantum dots prepared by three different precursor systems (**a**) P_4_(P-P). Reprinted with permission from Ref. [48]. Copyright 2017, American Chemical Society. (**b**) PH_3_(P-H). Reprinted with permission from Ref. [49]. Copyright 2018, Royal Society of Chemistry. (**c**) PCl_3_(P-Cl). Reprinted with permission from Ref. [51]. Copyright 2008, John Wiley and Sons.

The synthesis of InP quantum dots mainly mimics the classical preparation of CdS quantum dots in high boiling solvents at high temperatures to precisely regulate the size of InP quantum dots by balancing the reaction rates of nucleation and growth. Currently, synthetic studies of InP quantum dots have focused on precursor chemistry and core/shell structure engineering [45]. For more than 30 years, most syntheses of InP have been based on adaptations of dehalogenation silanation reactions and typically use P(SiMe_3_)_3_ as precursor [46]. However, P(SiMe_3_)_3_ is self-combustible and costly accounting for more than 90% of the total synthesis cost. In order to find a source of phosphorus with less spontaneous combustion, inorganic phosphorus was used instead. The dissociation energy of the P-P single bond in white phosphorus (P_4_) is approximately 460 kJ mol^−1^ slightly higher than that of the P-Si bond in P(SiMe_3_)_3_ (363 kJ mol^−1^) [47], which is an advantageous alternative precursor, which are advantageous alternative precursors. Bang and colleagues reported in 2017 the pioneering work of controlled synthesis of InP quantum dots from P4 (Figure 5a) [48]. The relatively low dissociation energies of P-H bonds (351 kJ mol^−1^) and P-Cl bonds (356 kJ mol^−1^), common intermediates in the phosphorus chemistry industry, make phosphine (PH_3_) and phosphorimide (PCl_3_) amenable to the preparation of InP quantum dots (Figure 5b,c), and limited success has been achieved with them [49,50,51]. Despite the economic value of the development of these precursors, they have not been widely used due to issues such as toxicity and poor control of the particle size distribution due to the fast reaction from low bond dissociation energies. Encapsulation of InP QDs with ZnSe and ZnS increases PL QY to 98%, reduces the capture PL to 13% of the total PL, and extends the double exciton lifetime by pushing the defects closer to the energy band edges [52,53]. ZnS is the main shell material because its wide band gap (3.54 eV) ensures strong confinement of the two exciton carriers. Unfortunately, the large lattice mismatch (7.8%) between ZnS and InP hinders thick epitaxial shell growth. Theoretically, InP/ZnS alloys, either in the whole particle or only at the interface, could modulate the lattice mismatch to reduce the strain at the core/shell interface and grow thicker ZnS shells with improved PL properties [54,55,56].

The preparation of InP quantum dots still requires stringent experimental conditions due to the high reactivity of P towards In and impurities such as water and oxygen. Therefore, continuous efforts are needed to refine the synthesis protocols as well as to find more suitable precursor materials. The synthesis of large InP nanocore shells surrounded by well-defined crystal structures is also expected to be established.

### 2.2. Group IV Quantum Dot Photocatalysts

Group IV quantum dots, which are mainly composed of group IV elements such as carbon or silicon, have been one of the research hotspots in recent years. In 2004, while studying the preparation of carbon nanotubes by arc discharge, Xu accidentally discovered for the first time small nanoparticles with fluorescent properties, which were later named as carbon quantum dots [57]. In 2004, Xu accidentally discovered small nanoparticles with fluorescent properties while studying the preparation of carbon nanotubes by arc discharge. Typically, carbon quantum dots refer to carbon materials with dimensions less than 10 nm, with a core–shell structure, usually with no obvious boundary between the carbon core and the functional group/polymer shell [58]. The carbon nuclei are amorphous. The carbon core is an sp2 hybridized carbon structure surrounded by an amorphous structure, while the functional group/polymer shell structure provides functionality [59]. The carbon quantum dots are a type of inorganic As an inorganic non-metallic nanomaterial, carbon quantum dots have no toxic effects and less environmental damage than the two materials introduced above; in addition, the luminescence mechanism of carbon quantum dots may be related to the carbon core, surface, and molecular states, whereas the luminescence mechanism of metallic quantum dots is attributed to the bandgap and the electronic jumps [60]. Due to the wide range of raw materials for the preparation of carbon quantum and the variety of preparation methods, carbon quantum dots with different structures and chemical compositions have been produced. Researchers are now beginning to pay more attention to the synthesis–structure–property relationships of carbon quantum dots with a view to optimizing the final properties.

## 3. Synthesis and Preparation of Quantum Dots

Precursors and synthesis methods together influence the structure and properties of quantum dots, leading to a diversity of quantum dots. Usually quantum dots are prepared in two ways: top-down and bottom-up methods. This section collates the usual preparation methods and advantages and disadvantages of quantum dot materials.

### 3.1. Top-Down Methods

Top-down methods involve starting with a large-sized material and physically or chemically cutting or stripping it down to quantum dot size. Top-down methods include epitaxial growth, mechanical ball milling, and electrochemical methods.

**Figure 6:**
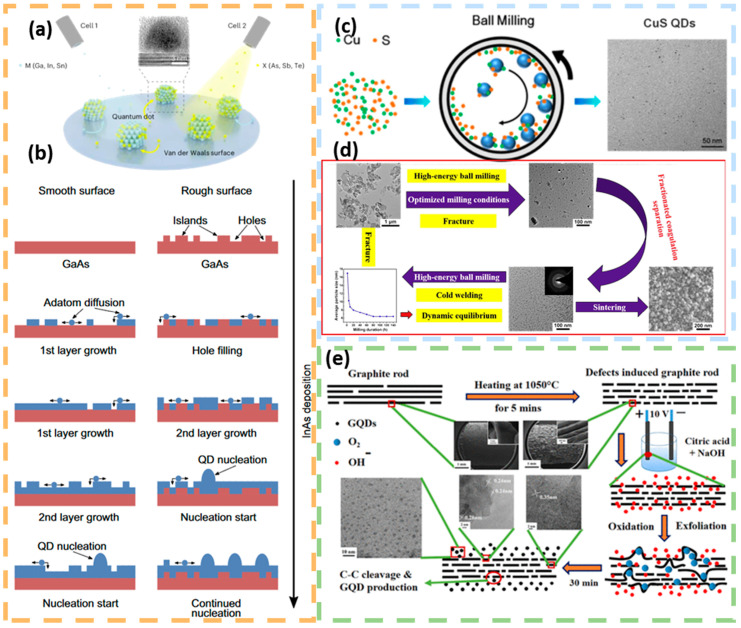
Preparation of quantum dots by top-down method (**a**,**b**) Different quantum dots were prepared by epitaxial growth method (**a**) Growing InSb quantum dots on a van der Waals surface. Reprinted with permission from Ref. [63]. Copyright 2024, Springer Nature. (**b**) Schematic illustrating InAs layer (blue) development under increasing InAs deposition on a smooth and rough GaAs surface (red). Adatom diffusion (blue dots) takes place on the surface. QD nucleation (blue domes) on rough surfaces starts earlier than on smooth surfaces. Reprinted with permission from Ref. [60]. Copyright 2022, Springer Nature. (**c**) Preparation of CuS quantum dots by mechanical ball milling. Reprinted with permission from Ref. [64]. Copyright 2016, Elsevier. (**d**) Preparation of Al_2_O_3_ quantum dots by mechanical ball milling. Reprinted with permission from Ref. [65]. Copyright 2018, Elsevier. (**e**) Under the action of electric field, carbon quantum dots are stripped from 2D graphite phases. Reprinted with permission from Ref. [66]. Copyright 2017, American Chemical Society.

The epitaxial growth method uses the stress generated by lattice mismatch as a driving force to self-coalesce epitaxial material deposited on the substrate surface through the deposition of single-crystal thin films prepared on a single-crystal substrate. This method can be used in conjunction with micro- and nanofabrication techniques to control the shape and density of quantum dots. The products prepared by the epitaxial growth method are of extremely high purity, and they grow quantum dots by periodically depositing materials such as InAs on the substrate [61]. The quality of the quantum dots is improved by depositing one or more buffer layers (e.g., GaAs and AlAs) on the substrate, which helps to reduce dislocations and strains (Figure 6b). After the quantum dots are grown, they are usually covered with one or more layers of material to protect the quantum dots [62]. Obviously, the quantum dot materials prepared by this method are highly pure and stable, but the preparation process is relatively complex and monodisperse quantum dots cannot be obtained. The size and density of quantum dots can be regulated by adjusting the substrate temperature and growth time, such as growing InAs quantum dots at different temperatures to obtain different densities of quantum dots (Figure 6a) [63].

The mechanical ball milling method is based on the interaction between the substrate powder and the milling balls during the ball milling process, where the raw material is continuously shaped and deformed, and then broken again after a certain degree, and finally milled into small particles. This method can obtain quantum dots with different particle sizes by optimizing parameters such as ball milling speed, ball–powder mass ratio and ball milling time, and is mostly used for laboratory-scale material preparation. Li et al. synthesized CuS quantum dots of approximately 5 nm using simple mechano-chemical ball milling (Figure 6c), which showed a strong absorption peak at 363 nm [64]. High-temperature calcined alumina powder was ball-milled to obtain α-Al_2_O_3_ quantum dots with an average particle size of 8 nm (Figure 6d) [65]. The average particle size of α-Al_2_O_3_ quantum dots was 8 nm. However, this approach is not suitable for large-scale production, and the ball-milled quantum dots may agglomerate and affect the performance.

The electrochemical stripping method uses electrolyte ions under the action of an electric field to destroy the physicochemical properties of the electrode surface and strip down small-sized nanocrystals. Electrochemical methods can be used to prepare carbon quantum dots, which have excellent properties such as very good dispersion, high chemical inertness, and low cytotoxicity in aqueous media. In 2004, Xu made the first accidental discovery of carbon quantum dots with fluorescent properties in an experiment to prepare carbon nanotubes by arc discharge [57]. Satyaprakash Ahirwar investigated the effect of acidity and alkalinity of electrolyte on the structure of quantum dots. Using alkali hydroxide and citric acid as the electrolyte, the oxygen-rich functional groups in the quantum dots increased with the increase in hydroxide concentration in the electrolyte to obtain graphene oxide quantum dots (Figure 6e) [66].

In summary, the preparation method of top-down quantum dots has the advantages of low cost of raw materials, simple steps and rapid preparation, but its synthesized quantum dots have the limitations of uneven size and shape distribution, surface defects and structural damages.

### 3.2. Bottom-Up Methods

Bottom-up methods refer to the gradual assembly of quantum dots by chemical reactions starting from the atomic or molecular level. Bottom-up methods include laser ablation, colloidal chemistry, organic thermal injection, and water/solvent thermal synthesis.

**Figure 7:**
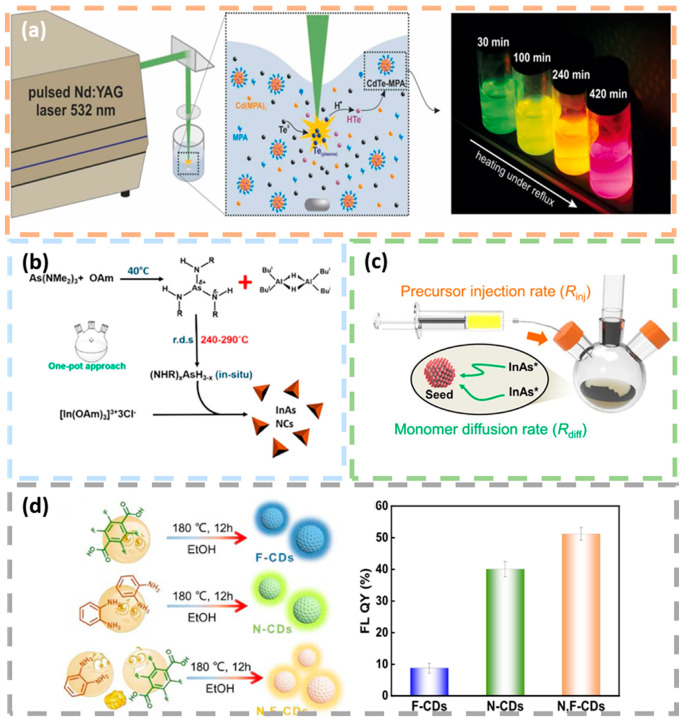
Preparation of quantum dots by bottom-up method (**a**) CdTe-MPA QDs production by laser ablation bottom-up assisted method. Reprinted with permission from Ref. [67]. Copyright 2022, Elsevier. (**b**) The mechanism regulating the formation of InAs QDs using DBAL-H as reducing agent. Reprinted with permission from Ref. [70]. Copyright 2022, Royal Society of Chemistry. (**c**) Colloidal InAs QD growth via continuous injection of InAs cluster-based single-source precursor into InAs seed solution. Reprinted with permission from Ref. [72]. Copyright 2021, Springer Nature. * is just adnotation. (**d**) Schematic illustration of the design and synthesis of F-CDs, N-CDs and N, F-CDs and comparison of FL QY of three prepared CDs. Reprinted with permission from Ref. [76]. Copyright 2023, Elsevier.

Laser ablation involves irradiating a target material with a high-power laser beam, resulting in rapid heating and evaporation of the material, leading to the formation of a plasma. The atoms, molecules and clusters created in this process can subsequently form quantum dots. In the case of carbon-based quantum dot materials, for example, the carbon-based target typically transforms into a graphite-like phase structure when exposed to the laser beam. This method prepares carbon quantum dots through a process of excision, decomposition and gasification of the carbon source. Laser ablation synthesis offers significant advantages in the preparation of highly crystalline and monodisperse quantum dots (Figure 7a) [67]. In 2006, Sun et al. mixed graphite powder with cement and pressed to prepare a carbon target [68]. This carbon target was laser ablated in the presence of water vapor and argon was used as a carrier gas to synthesize CQD. The resulting CQD showed a spherical structure with a diameter of 5 nm and a quantum yield of 4–10%. In 2008, Hu et al. [69] simplified the synthesis steps employed by Sun by combining laser ablation and passivation in one step, allowing the preparation of CQD with different surface coating conditions and different fluorescence emission wavelengths by varying the organic solvent only. One of the main advantages of utilizing laser ablation for the synthesis of CQD lies in its ability to produce highly crystalline, uniformly dispersed quantum dots. However, it is evident that specialized laser equipment and organic solvents or strong acid environments are complex and time-consuming to operate, and that quantum yields are low and expensive.

Colloidal chemistry prepares quantum dots by heating compound precursors in solution, often using organic ligands or surfactants to precisely control the size, shape and surface properties of the quantum dots. Quantum dots obtained by this method are usually dispersed in solution to form a colloidal solution. In 1983, Louis Brus demonstrated that changing the size of a cadmium sulfide colloid resulted in a change in its exciton energy, and for the first time, he introduced the concept of a colloidal quantum dot (QD) [35]. This work confirmed the concept of quantum dots and laid the foundation for subsequent research on quantum dot synthesis and applications. Earlier colloidal chemical methods synthesized quantum dots with poor crystallization and low luminescent quantum yields. By increasing the reaction temperature and using more effective surface passivators, researchers can improve the crystallization degree and luminescence efficiency of quantum dots. Jalali, H.B. et al. synthesized InAs colloidal quantum dots via a two-step synthesis of aminoarsenic as an arsenic precursor and aminophosphine as a reducing agent (Figure 7b) [70]. The InAs quantum dots prepared by introducing a reducing agent were state-of-the-art in terms of size dispersion and band gap range.

Organic thermal injection, in which the nucleation and growth of quantum dots is promoted by rapidly injecting the precursor into a high temperature organic solvent, usually in an inert atmosphere, was first proposed by Murray, C.B.‘s team in 1993 for the preparation of nearly monodisperse semiconductor nanocrystals of CdE (E = S, Se, Te, i.e., cadmium sulfide, cadmium selenide, cadmium telluride) by high temperature pyrolysis, and these nanocrystals are also now known as quantum dots [71]. The publication of this method has made it possible for the first time to synthesize specific quantum dots. The publication of this method made it possible for the first time to synthesize quantum dots of a specific size. The synthesis of uniformly sized quantum dots by continuous injection is now well established. In the synthesis of quantum dots above 5 nm particle size, secondary nucleation or particle growth during growth is accompanied by size inhomogeneity. Kim et al. made a breakthrough in controlling the diffusion kinetics by adjusting the reaction volume, the concentration of the precursor, and the rate of precursor injection (Figure 7c) [72], synthesized InAs quantum dots with a size larger than 9.0 nm and a narrow size distribution (12.2%). The diffusion kinetics-controlled synthesis overcomes the size limitation during particle growth due to reactive monomers. Inspired by this work, Liu et al. [73] achieved a narrowing of the size distribution of CdS colloidal quantum dots at high monomer concentrations. By using cadmium carboxylate ligands, CdS colloidal quantum dots can grow into both cubic and irregular hexahedral morphologies. By combining the modulation of monomer concentration and reaction temperature, CdS colloidal quantum dots with sizes ranging from 4 to 7 nm (fluorescence peaks from 470 to 500 nm) and half-peak full widths of fluorescence spectra of only 75 meV were successfully synthesized. It was found that the broadening of the quantum dot size was a result of different growth rates of quantum dots with different morphologies, and the different growth rates were caused by different ligand permeability.

Hydrothermal/solvothermal method is a rapid synthesis method to synthesize quantum dots under high temperature and pressure water/solvothermal conditions. The hydrothermal/solvothermal method is widely used for one-step synthesis of quantum dots by adding one or more precursor materials to a solvent, and the precursor materials are co-nucleated and shaped in a confined high-temperature, high-pressure environment. In 2010, Zhang’s group pioneered the use of one-step hydrothermal method to produce carbon quantum dots [74]. Using ascorbic acid as a precursor and ethanol as a solvent, the reaction was carried out at 180 °C for 4 h. After extraction with dichloromethane and dialysis with a cellulose acetate dialysis membrane, carbon quantum dots consisting of disordered graphene and amorphous structures with a particle size of approximately 1.2 nm were obtained. The fluorescence quantum yield of these carbon quantum dots was 6.79%. Researchers have now begun to use this method to fabricate doped carbon quantum dots for different experimental requirements.Hu used a one-pot solvothermal strategy to investigate the optical properties of nitrogen-doped carbon quantum dots prepared from four precursors with different nitrogen contents [75]. Hua improved the synthesis pathway and synthesized nitrogen and fluorine co-doped tunable luminescent quantum dots with a quantum yield of 52.2% and multicolor tunable luminescence from blue to red, using o-phenylenediamine and tetrafluoroterephthalic acid as raw materials (Figure 7d) [76].

In summary, the bottom-up method can more precisely control the composition, size and shape of quantum dots, with narrow size distribution and flexible surface modification. Comparatively, most bottom-up methods, which require precise control of process parameters such as reaction temperature, time, and pressure have complex operation steps and require additional separation and purification steps, low synthetic yields, and poor stability of the synthesized quantum dots. Therefore, the selection of a suitable preparation method requires comprehensive consideration based on specific application requirements, cost-effectiveness and sustainability.

## 4. Mechanism of Microbial Corrosion Resistance of Photocatalytic Quantum Dots

Quantum dots have gained wide attention in the antibacterial field due to their stable structure, photoluminescence, adjustable size and easy surface modification. Photocatalytic quantum dots material generates reactive oxygen species under light, which binds with the DNA strand of bacteria, hinders DNA replication and bacterial division and proliferation, destroys the normal life activities of bacteria, and prompts the death of bacteria.

**Figure 8:**
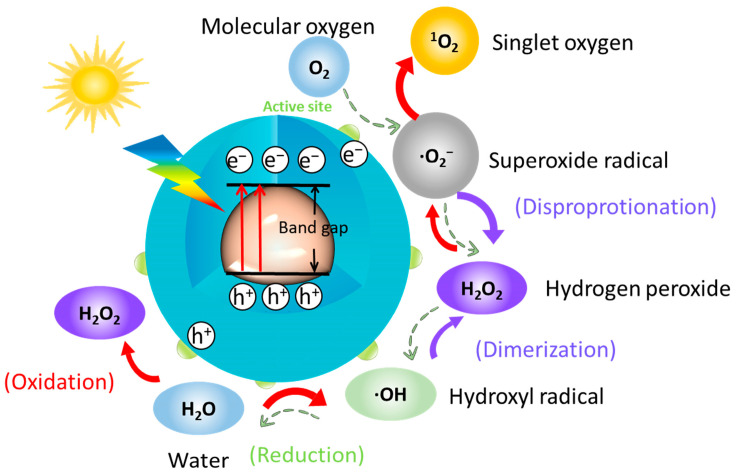
Gradual production of reactive oxygen species by quantum dots under photocatalysis.

Recently, quantum dots have shown very strong potential for photodynamic therapy due to their excellent photoluminescent properties and very wide visible light absorption range from the UV to the NIR and have been reported for cancer treatment [77,78,79]. Similarly, quantum dot materials can produce reactive oxygen species upon photoexcitation to kill or inhibit bacteria, and the redox potentials of some of the reactive oxygen species are shown in Table 1. Existing findings suggest that the photochemical structure of quantum dots can be used for effective charge separation, where electrons are excited from the valence band to the conduction band and leave holes in the valence band [80]. Subsequent transfer of excited electrons to oxygen produces O_2_^−^ and ·OH, while the highly reactive holes react with water to produce ·OH [81,82]. The production of this class of reactive oxygen species is based on the transfer of electrons to oxygen. Gradual production of reactive oxygen species by quantum dots under photocatalysis are shown in Figure 8. In addition, several studies have shown that quantum dots can produce ^1^O_2_. The process of photoinduced energy transfer is related to the process of photoinduced electron transfer due to the coexistence of excitons and carriers. Single heavy state excitons can be formed from a pair of electrons at the CB minimum and holes at the VB maximum as a result of energy release during the binding process. Based on the Saha–Langmuir equation, the concentration ratio of excitons to free charge carriers (temperature and photon excitation density) under certain conditions is mainly determined by the exciton binding energy [83]. Excited singlet excitons (S) are then transferred to the long-lived triplet state (T) via an intersystem crossing process, leading to the production of molecular oxygen in the ground state via energy transfer ^1^O_2_ [84]. Under light conditions, quantum dots are prone to produce reactive oxygen species such as superoxide and hydroxyl radicals, which damage bacterial DNA and proteins [85].

**Figure 9:**
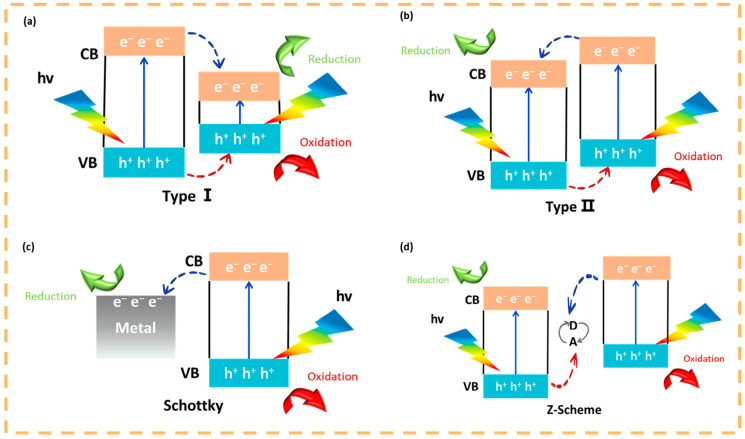
Schematic diagram of four photocatalytic heterojunction mechanisms: (**a**,**b**) Type Ⅰ and Ⅱ of p-n junction; (**c**) Schottky junction; (**d**) Z-scheme junction.

**Figure 10:**
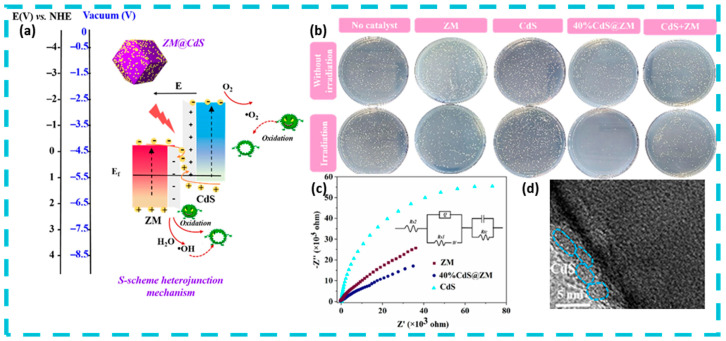
Photocatalytic mechanism and properties of S-scheme junction. Reprinted with permission from Ref. [88]. Copyright 2022, Elsevier. (**a**) S-scheme transfer mechanism of CdS@ZM nanocomposites under visible light irradiation. NHE, normal hydrogen electrode. (**b**) Antibacterial effects with and without illumination of the blank experiment and ZM, CdS, and CdS@ZM nanocomposites. (**c**) Electrochemical impedance spectra of ZM, CdS, and the 40% CdS@ZM composite in a 0.5 mol/L Na_2_SO_4_ electrolyte. (**d**) Transmission electron microscopy images of the used 40%CdS@ZM.

As mentioned earlier, elemental doping, heterostructure building can enhance the photocatalytic activity of quantum dots, and doping of elements N and S broadens the light absorption limit of quantum dot materials to the near-infrared, with N introducing extra electrons, and S doping introducing extra holes to reduce photogenerated carrier complexation [86,87]. Photocatalytic heterojunctions are generally classified into three types, p-n heterojunctions, Schottky heterojunctions and Z-type heterojunctions. p-n heterojunctions contain two types of mechanisms, Type I (Figure 9a) heterojunctions are those in which the conduction (CB) and valence bands (VB) of one semiconductor are situated between those of another semiconductor leading to the accumulation of carriers in the low-bandgap material, and it is generally accepted that type I heterojunctions are relatively inefficient in charge separation. Type II (Figure 9b) heterojunction where both CB and VB of one semiconductor are higher than the other semiconductor, this structure facilitates the transfer of electrons from the high CB material to the low CB material and their accumulation in the low CB material, while holes are transferred from the low VB material to the high VB material and their accumulation in the high VB material. The Schottky heterojunction (Figure 9c) is a semiconductor-metal structure, where electrons are transferred into the metal and a Schottky barrier is formed at the interface. The Z-type heterojunction (Figure 9d) introduces a new intermediate electron transferor on the basis of the type-II heterojunction, where some of the electrons from the high-CB material and some of the holes from the high-VB material are transferred into the electron transferor, with the same over-sacrifice of a small number of carriers in order to obtain a large number of charge Liang et al. designed excellent stepped (S-scheme) CdS@ZM heterojunctions by in situ coating CdS quantum dots (QDs) on zeolite imidazolium ester dodecahedral skeletons (ZMs), and the S-type CdS@ZM composites exhibited excellent interfacial contacts and uniform morphological distribution. Under visible light irradiation (λ ≥ 420 nm), the 40% CdS@ZM heterojunction achieved 97.6% disinfection and bactericidal rate against Staphylococcus aureus within 15 min (Figure 10) [88]. Based on its photocatalytic performance superior to that of type II heterojunction and combining the energy band structure, internal electric field and ESR spectroscopic data, a tentative photocatalytic mechanism was proposed, in which CdS and ZM generate electron–hole pairs under light, and under the action of the internal electric field, the electrons in the ZM conduction band that are not involved in the reaction are transferred and recombined with the holes in the CdS valence band that are not involved in the reaction, which contributes to the ZM conduction band and the CdS valence band having enhanced redox potential. Under the enhanced redox potential, ·OH and ·O_2_^−^ reactive substances are produced that are sufficient to react with *S. aureus*.

**Figure 11:**
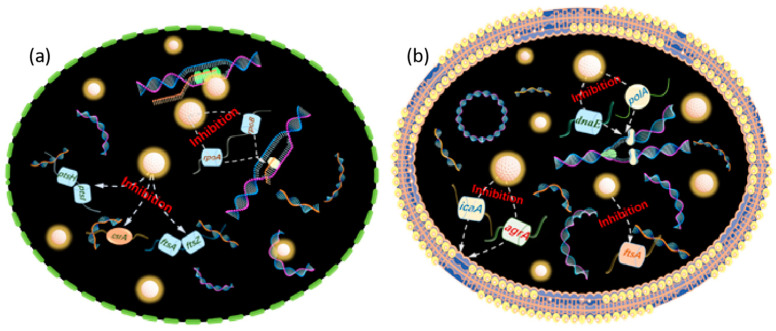
Schematic diagram of antibacterial mechanisms of N, F-CDs against *E. coli* (**a**) and *S. aureus* (**b**) at molecular level. Reprinted with permission from Ref. [76]. Copyright 2023, Elsevier.

**Figure 12:**
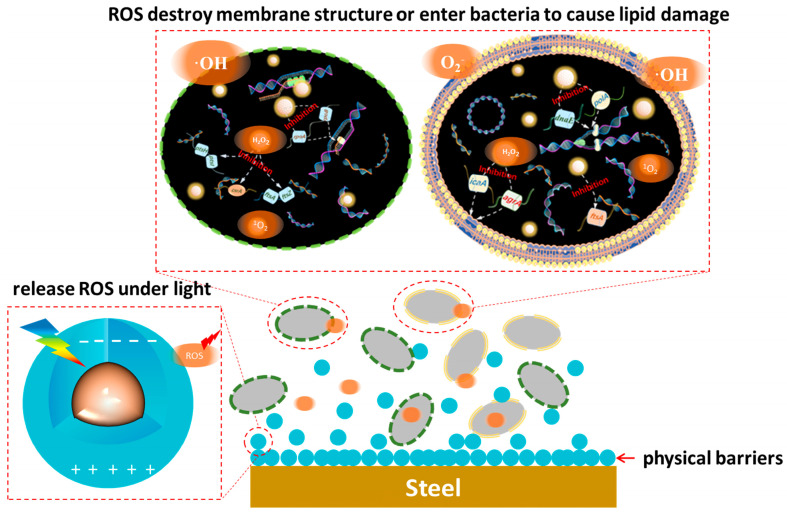
Photocatalytic corrosion prevention mechanism of quantum dots in light. Reprinted with permission from Ref. [76]. Copyright 2023, Elsevier.

The mechanism related to the antibacterial properties of ROS substances has been quite well done in previous work, reacting with genetic material, enzymes, proteins, etc., within bacteria to cause oxidative damage, or reacting with structural components of the cell membrane (wall) to cause lipid peroxidation of the cell, resulting in the damage and death of the bacteria [89,90] The bacterial damage can result in death. Specifically, ROS substances such as ·OH and O_2_^−^, which cannot cross the cell membrane, inactivate lipids mainly by inducing oxidative reactions, thus causing bacterial death [91,92]. As oxidant, ·OH reacts with C=C bonds on cell membrane lipids to produce peroxides or oxidative molecules, and it can even act on fungal cell walls to transform glycosidic bonds into ester bonds, destroying the integrity of the cell wall. O_2_^-^ is difficult to penetrate through negatively charged phospholipid bilayers due to its hydrophilic electronegativity, but it can disproportionate to H_2_O_2_ under the action of superoxide dismutase or be converted to HOO· by H^+^ and accumulate in large quantities in the hydrophobic region of the cell, leading to the death of lipids. hydrophobic region, triggering lipid peroxidation. Diffusible small molecules such as H_2_O_2_ can enter into bacteria and react with proteins and enzymes to hinder normal physiological functions; ^1^O_2_ attack amino acids and ribonucleotides to cause irreversible oxidative damage to proteins and DNA (or RNA), which ultimately leads to apoptosis [93,94]. Reactive oxygen species can co-exist and can be transformed into each other. Interestingly, free quantum dots in aquatic environments as zero-dimensional nanomaterials with a size of less than 10 nm can also pass through the phospholipid bilayer and enter the bacterial interior, where they bind to DNA strands. It hinders asexual proliferation and genetic information transfer, inhibits biofilm formation, and triggers bacterial apoptosis (Figure 11 and Figure 12) [76].

In addition, quantum dot materials can contribute a certain physical barrier effect by physically or chemically hindering microorganisms from attaching to the surface of the material and inhibiting biofilm formation. Most corrosion inhibitors usually also have atoms such as S, O, and N, which physicochemically and chemically adsorb the corrosion inhibitor molecules to the metal surface by providing lone pair electrons to interact with the empty metal orbitals to form ligand bonds [95,96]. Quantum dots such as ZnO, CdS, TiO_2_, CuInS_2_, Ag-QDs, and CQDs have a large number of unsaturated bonds and reactive functional groups on the surface, and these reactive sites interact with the metal substrate to form complexes, which comprise a thick film and act as a physical barrier [97]. Transition group metal oxide quantum dots such as ZnO and TiO_2_ quantum dots can form coordination bonds with empty metal orbitals through the oxygen atoms on the surface of the quantum dots and further act as precursors to produce a hydroxide protective film under the action of the medium [9]. Metal sulfide quantum dots reduce bacterial adsorption by competing with bacteria for active sites on the metal surface to form complexes through S^2−^ complexation [98]. Carbon quantum dot materials form coordination bonds with metals through surface-enriched unsaturated functional groups (e.g., hydroxyl groups, carboxyl groups, carbon-carbon double bonds), and with the formation of the retardation layer, the contact angle of the metal surface increases and hydrophobicity is enhanced [99]. When in contact with bacteria, quantum dots can physically or chemically interact with the bacterial membrane, altering the bacterial membrane structure, causing the bacteria to rupture and die or detach from the material surface [100,101,102]. The adsorption of quantum dots on bacteria and the ability to hinder their attachment to the metal matrix greatly shortened the propagation distance of active oxygen species from quantum dots to bacteria, effectively avoided the defects of short active oxygen species effective period and poor stability, and greatly enhanced the antimicrobial ability of quantum dot photocatalysts.

## 5. Challenges and Future Prospects of Quantum Dots in Photocatalytic Antimicrobial Protection

Photocatalytic quantum dots in the field of microbial corrosion resistance, although showing great potential, still have some challenges and limitations: The traditional II–VI group quantum dots represented by CdS are biotoxic, which is destructive to the ecology of certain application environments. The III–V quantum dots such as InP, which are their potential substitutes, still have a considerable gap in the synthesis route and catalytic performance. Photocorrosion may occur in quantum dot materials under prolonged light exposure, such as CdS quantum dots, resulting in reduced photocatalytic activity or even protection failure. To a certain extent, it increases the maintenance cost and reduces the service life, which affects its use value to a certain extent.

The photosensitive material-microorganism interface determines the electron transfer efficiency, and the quantum efficiency serves as one of the important evaluation indexes for light-driven microbial hybrid systems. In this process of electron transfer, some quantum dots suffer from low quantum efficiency, which greatly reduces the antimicrobial efficiency of quantum dot materials.

To address the above challenges, future research can explore the following directions: Atomic-scale analysis: a large number of studies and modifications have been reported on the photocatalytic properties of quantum dots, but the current research on the composition–structure–property relationship at the atomic scale of quantum dots is not systematic enough to be perfect. An in-depth study of the structure-property relationship of quantum dot materials at the atomic scale is essential for the development of new, efficient and functionalized quantum dot materials.

Structure construction: As special zero-site nanomaterials, the unique size effect of quantum dots plays an important factor in their photocatalytic antimicrobial properties. Currently, the ways to enhance the antimicrobial performance of quantum dots are mainly focused on the precise size modulation, functionalized functional groups or elemental doping, and the construction of special core–shell heterostructures. All of these approaches are practical and effective, but there is no scientific intrinsic connection between them. The ratio control of the core–shell structure, the different mechanisms of functionalized functional groups or elemental doping inside and outside the nucleus of quantum dots is one of the hot spots for future research.

Development of electron mediators and transfer bodies: studies on the efficiency of electron transfer between photosensitized materials and biological interfaces intuitively affect the efficiency of quantum dot materials in antimicrobial applications. The selection or development of more efficient electron mediators to improve the electron transfer efficiency from photosensitizers to microorganisms, as well as the development of effective electron transfer bodies to support the further transfer of electrons across cell membranes, play a guiding role in the development of advanced photocatalytic antimicrobial quantum dot nanomaterials.

Targeted sterilization: Current antimicrobial protection means are mostly simple and brutal to kill all bacteria, however, not all bacterial activities are harmful to the corrosive behavior of metal materials under the practical application of metal materials. Based on the targeting properties of antibiotics and other medical sterilizing substances, it is a novel and bright direction of development to construct suitable quantum dot materials against a specific type of bacteria.

To sum up, there are some difficulties in the application of photocatalytic quantum dot materials, such as unstable quantum yield, imprecise structure control and poor electron transfer efficiency. Therefore, the structural development and special modification of quantum dot materials (such as higher electron transfer efficiency and stronger targeting selection) are promising and necessary.

## 6. Conclusions

Because of its unique nanostructure and quantum effect, quantum dot materials have shown great potential in the field of photocatalytic antimicrobial and microbial corrosion resistance. These semiconductor nanomaterials not only have a wide light absorption range from ultraviolet to near-infrared and high photocatalytic activity, but also provide a new option for traditional fungicides as an environmentally friendly alternative. By forming protective films and intelligent coatings, quantum dots provide basic protection at the physical and chemical level for metal materials, and can also develop self-repairing and self-warning coating systems with their fluorescence characteristics to monitor the coating status in real time.

In terms of photocatalytic antibacterial, quantum dots can produce reactive oxygen species (ROS) under light, which can damage the DNA and proteins of bacteria, thereby inhibiting their growth and reproduction. In addition, the surface modifiers of quantum dots enable them to hinder the adhesion of microorganisms on the surface of the material by physical or chemical means, inhibiting the formation of biofilms, thus providing an additional physical barrier effect. In general, photocatalyzed quantum dot materials provide a new idea for the field of microbial corrosion resistance.

## Figures and Tables

**Table 1:** Standard redox potentials of selected reactive oxygen species.

Half Reaction	Electrode Potential/V
·OH + e^−^ + H^+^ → H_2_O	+2.31
O_3_ + 2e^−^ + 2H^+^ → H_2_O + O_2_	+2.075
H_2_O_2_ + 2e^−^ + 2H^+^ → 2H_2_O	+1.76
·RO + e^−^+ H^+^ → ROH	+1.6
·HO_2_ + e^−^ + 2H^+^ → H_2_O_2_	+1.06
·ROO + e^−^ + H^+^ → ROOH	+1.0
^1^O_2_(g) + e^−^ → ·O_2_^−^	+0.64
H_2_O_2_ + e^−^ + H^+^ → H_2_O +·OH	+0.32
·O_2_^−^ + e^−^ + 2H^+^ → H_2_O_2_	+0.36
O_2_(aq) + e^−^→ ·O_2_^−^	−0.33
H_2_O + e^−^ → e_aq_^−^	−2.87

## Data Availability

Data are available upon request.
